# An artificial intelligence model for electrocardiogram detection of occlusion myocardial infarction: a retrospective study to reduce false-positive cath lab activations

**DOI:** 10.1093/ehjdh/ztaf138

**Published:** 2025-12-02

**Authors:** Benjamin L Cooper, Evan A Genova, Carrie A Bakunas, Catherine E Reynolds, Benjamin Karfunkle, Nils P Johnson

**Affiliations:** Department of Emergency Medicine, Memorial Hermann Hospital—Texas Medicine Center, UTHealth Houston, McGovern Medical School at UTHealth Houston, 6431 Fannin St., JJL 260, Houston, TX 77030, USA; Department of Emergency Medicine, Memorial Hermann Hospital—Texas Medicine Center, UTHealth Houston, McGovern Medical School at UTHealth Houston, 6431 Fannin St., JJL 260, Houston, TX 77030, USA; Department of Emergency Medicine, Memorial Hermann Hospital—Texas Medicine Center, UTHealth Houston, McGovern Medical School at UTHealth Houston, 6431 Fannin St., JJL 260, Houston, TX 77030, USA; Department of Emergency Medicine, Memorial Hermann Hospital—Texas Medicine Center, UTHealth Houston, McGovern Medical School at UTHealth Houston, 6431 Fannin St., JJL 260, Houston, TX 77030, USA; Department of Emergency Medicine, Memorial Hermann Hospital—Texas Medicine Center, UTHealth Houston, McGovern Medical School at UTHealth Houston, 6431 Fannin St., JJL 260, Houston, TX 77030, USA; Department of Emergency Medicine, Memorial Hermann Hospital—Texas Medicine Center, UTHealth Houston, McGovern Medical School at UTHealth Houston, 6431 Fannin St., JJL 260, Houston, TX 77030, USA

**Keywords:** ST-elevation myocardial infarction, Occlusion myocardial infarction, Electrocardiogram, AI, PMcardio, Queen of Hearts

## Abstract

**Aims:**

Existing ST-segment elevation myocardial infarction (STEMI) alert pathways that rely on traditional STEMI criteria perform suboptimally. We aimed to evaluate the diagnostic performance of an artificial intelligence (AI) model to detect acute occlusion myocardial infarction (OMI) from the routine 12-lead electrocardiogram (ECG) and, specifically, its potential to reduce false-positive activations.

**Methods and results:**

Consecutive adults managed via the STEMI pathway were included from a tertiary academic medical centre between January 2022 and December 2023. Cases without an available ECG for review, death prior to catheterization, or alternative reasons for activation (i.e. electrical instability or urgent interventions) were excluded. Pre-coronary angiography tracings were interpreted via the AI tool. Test characteristics were compared against traditional STEMI criteria. The primary outcome was the number of avoidable false-positive activations. During the 2-year study period, there were 454 activations, 150 were excluded, and 304 cases with unique ECGs were included in the study cohort. There were 118 (38.8%) false-positive activations, of which 86 (72.9%) were correctly predicted by the AI model. Its test characteristics for identifying true positives were superior compared with traditional STEMI criteria for a sensitivity of 89.2% [95% confidence interval (CI): 84.0–92.9] vs. 68.3% (95% CI: 61.3–74.5), specificity 72.9% (95% CI: 64.2–80.1) vs. 51.7% (95% CI: 42.8–60.5), and accuracy 82.9% (95% CI: 78.3–86.7) vs. 61.8 (95% CI: 56.3–67.1).

**Conclusion:**

The AI model is superior to traditional STEMI criteria for detecting OMI and has the potential to reduce false-positive catheterization lab activations. It can be a useful decision-aid for catheterization lab activation.

## Graphical Abstract

**Graphical Abstract:**
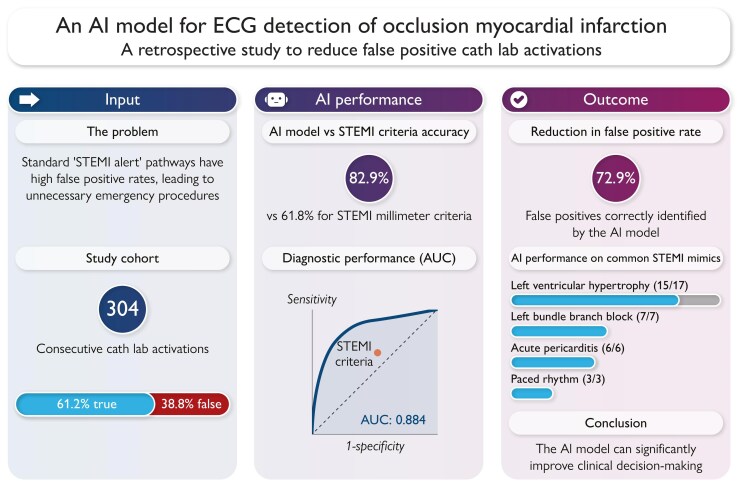

## Introduction

Chest discomfort is the most common primary symptom among patients presenting to the emergency department, accounting for 15–20% of all visits.^1^ For such patients, a 12-lead electrocardiogram (ECG) is obtained within 10 min of presentation to detect acute coronary occlusion and activate an emergent reperfusion therapy protocol, usually with primary percutaneous coronary intervention (PCI) or alternatively intravenous thrombolytic therapy. This emergent pathway, standardized worldwide, is primarily facilitated by detecting ST-segment elevation myocardial infarction (STEMI) criteria, generally known as ‘STEMI Alert’ pathways.^2,3^

However, existing STEMI alert pathways are suboptimal in performance. In systems where the first point of contact triggers the STEMI alert (either emergency medicine or paramedics), up to 40% are reported to be false positives,^4–6^ and the cardiology cancellation rate has been reported as high as 60%.^7^ Conversely, cardiology-led emergent cardiac catheterization laboratory (CCL) activation significantly impacts door-to-balloon (D2B) times, the most important determinant of both short- and long-term survival in patients with acute coronary occlusion.^8^ While the prevailing paradigm relies on the accuracy of the STEMI criteria for diagnosing occlusion myocardial infarction (OMI),^9^ de Alencar Neto *et al.*^10^ found that the pooled sensitivity of the STEMI criteria was only 43.6%, Khan *et al.*^11^ reported that over one quarter of patients diagnosed with a non-NSTEMI had acute total occlusion, and a 2018 meta-analysis of over 60 000 patients with NSTEMI reported the proportion of an occluded culprit artery to be 34%.^12^ Consequently, a method for improving the OMI detection using the ECG is needed.

Recently, artificial intelligence (AI) has been proposed as a potential alternative solution. A deep neural network (DNN) model has been developed to detect STEMI and STEMI-equivalent ECG patterns consistent with angiographically confirmed acute coronary occlusion from any 12-lead tracing, regardless of the format or device vendor. In external, prospective implementations, the model achieved a reduction in false-positive CCL activations and time to primary PCI,^13,14^ and displayed test characteristics superior to STEMI millimetre criteria.^15–17^

The current study evaluated the potential efficacy of the AI model in reducing the number of false-positive emergent CCL activations in an independent, retrospective cohort of consecutive patients activated via the existing ‘STEMI Alert’ pathway at a single centre. Ultimately, the project sought to determine the potential benefits of integrating advanced diagnostics, such as AI-powered ECG analysis, to enhance decision-making accuracy.

## Methods

Pre-coronary angiography (CAG) tracings were collected from the electronic medical record for consecutive CCL activations during a 2-year period. In the case of multiple pre-CAG recordings, the investigators (B.L.C., E.A.G., C.A.B., C.E.R., B.K.) determined via chart review of the ECG, which mostly influenced the activation decision. True-positive activations were identified using accepted angiographic criteria, including any records of intravascular imaging, how the lesion responded to wiring, and collateral maturation to distinguish chronic total or subtotal lesions from acute culprits. An interventional cardiologist blinded to AI model outcomes (but not the ECG) adjudicated equivocal cases (N.P.J.). True positives were defined as an acute culprit lesion with either (i) thrombolysis in myocardial infarction (TIMI) flow grade of 0–2 and any positive troponin, or (ii) TIMI flow grade of 3 and a very high peak troponin elevation (hs-cTnT ≥ 1000 ng/L, hs-cTnI ≥ 5000 ng/L, cTnI of > 10.0 ng/mL, or cTnT of > 1.0 ng/mL).^18–20^

The data analysis partner (Powerful Medical) provided per-patient AI interpretation for all ECGs by applying their model to the digitized ECG waveforms in batch mode but blinded to all clinical and angiographic results. The ECG digitization methodology has been described elsewhere.^21^ All ECGs were analysed using the CE-certified PMcardio platform with the Queen of Hearts AI ECG model (aOMI v1). Briefly, this DNN-based model has been trained to detect angiographically confirmed STEMI or STEMI equivalents regardless of the presence of STEMI millimetre criteria. Test characteristic confidence intervals were determined using the Wilson score. Test methods were compared using McNemar’s test for paired data. Data were managed and analysed using Microsoft Excel 365 (Microsoft Corporation, Redmond, WA, USA) and R version 4.4.3 (R Core Team, Vienna, Austria).

### Study design, setting, and population

The population included consecutive adult patients for whom an STEMI alert or CCL activation was triggered from January 2022 through December 2023. Individuals were included if the following criteria were met: (i) at least 18 years of age, (ii) at least one standard 10 s 12-lead ECG was available, and (iii) managed via the standardized ‘STEMI Alert’ pathway. Individuals were excluded if they (i) were managed via an acute coronary syndrome (ACS) pathway other than the ‘STEMI Alert’ CCL activation protocol, (ii) presented for an urgent cardiac catheterization (e.g. transfers for high-risk PCI or mechanical circulatory support), (iii) died prior to catheterization, (iv) received urgent or emergent catheterization for electrical instability outside of the context of suspected STEMI on electrocardiography (including cardiac arrest or ventricular storm), or (v) lacked a valid standard 10 s 12-lead ECG for which decisions were made (when performing chart review). In our institution, emergency physicians can activate the CCL unilaterally, and traditional STEMI millimetre criteria are not a precondition. The cardiologist reserves the right to cancel the activation, although this is a rare occurrence.

The estimated sample size was 200–300 patients based on our institutional volume. To ensure the robust statistical analysis, the sample size for this project was calculated based on an expected AI model sensitivity of 100% and specificity of 85% (68% reduction of false positives compared with standard of care), with a true STEMI prevalence of 0.75 among patients activated via the ‘STEMI Alert’ pathway. Accounting for a 10% dropout rate due to the unavailability of data, a total of 218 patients would achieve a confidence level of 95% and precision of ±0.10 for the specificity metric.

### Outcome measures

The primary endpoint was false-positive CCL activations comparing the AI model vs. routine clinical care. Secondary endpoints included:

- Sensitivity and specificity of AI model in detecting acute OMI
- Diagnostic accuracy of the AI model vs. traditional STEMI millimetre criteria^9^ in an acute OMI
- Total number of STEMI activations
- Total number of cancelled STEMI activations
- Total number of true OMI cases
- Total number of false-positive STEMI activations

## Results

**Figure 1:**
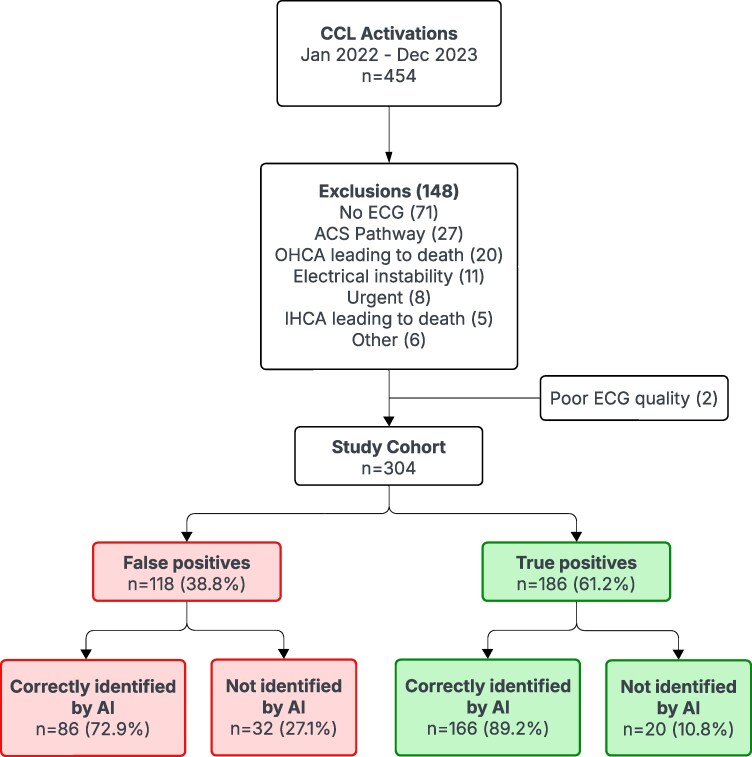
Flow chart. CCL, cardiac catheterization laboratory; ACS, acute coronary syndrome; OHCA, out-of-hospital cardiac arrest; IHCA, in-hospital cardiac arrest; AI, artificial intelligence.

During the 2-year study period, there were 454 CCL activations, of which 148 met exclusion criteria, 2 were excluded because of poor tracing quality (i.e. the AI model was unable to digitize the image to allow for interpretation), and 304 cases with unique ECGs were included in the final study cohort. A representative sample of digitized ECGs can be found in the supplementary content (see Supplementary material online, *Figure S1*). The mean age for the study cohort was 60 ± 13 years and 75% were male (*Table 1*). There were 118 (38.8%) false-positive activations, of which 86 (72.9% of false positives and 28.3% of the total study cohort) were correctly predicted by the AI model (*Figure 1*), and 186 true positives (61.2%), of which 166 (89.2%) were correctly identified by the AI model. Five of the total 304 CCL activations were cancelled by cardiology (1.6%)—four were ruled out via troponin (i.e. false-positive activations), and one was diagnosed with OMI via delayed catheterization (i.e. true-positive activation).

**Table 1:** Baseline demographics, angiographic findings, and interventions

Characteristic	Total cohort (*n* = 304)	True positive (*n* = 186)	False positives (*n* = 118)	*P*-value
Demographics				
Age, mean ± SD (years)	60 ± 13	59.7 ± 13.4	59.3 ± 13.6	0.813
Sex, *n* (%)				
Male	230 (75.7)	149 (80.1)	81 (68.6)	0.023
Female	74 (24.3)	37 (19.9)	37 (31.4)	0.023
Arrival method, *n* (%)				
EMS	172 (56.6)	115 (61.8)	57 (48.3)	0.020
Transfer	63 (20.7)	39 (21.0)	24 (20.3)	0.895
Inpatient	35 (11.5)	16 (8.6)	19 (16.1)	0.046
Walk-in	34 (11.1)	16 (8.6)	18 (15.3)	0.073
Cardiac arrest				
Arrest prior to angiography	53 (17.4)	24 (12.9)	28 (23.7)	0.015
Angiographic findings				
Culprit artery, *n* (%)	191 (62.8)	186	5 (4.2)	
LAD (including diagonal)	85 (44.5)	81 (43.5)	4 (3.4)	
LCx (including marginal)	14 (7.3)	14 (7.5)	0	
RCA	67 (35.1)	67 (36.0)	0	
Left main	4 (2.1)	3 (1.6)	1 (0.8)	
Multi-vessel	19 (9.9)	19 (10.2)	0	
Other	2 (1.0)	2 (1.1)	0	
TIMI flow grade, *n* (%)				
TIMI 0	123 (64.4)	123 (66.1)	0	
TIMI 1	28 (14.7)	26 (14.0)	2 (1.7)	
TIMI 2	27 (14.1)	26 (14.0)	1 (0.8)	
TIMI 3	13 (6.8)	11 (5.9)	2 (1.7)	
Intervention type, *n* (%)				
PCI	192 (63.2)	174 (93.5)	18 (15.3)	<0.001
CABG	15 (4.9)	3 (1.6)	12 (10.2)	<0.001
POBA	6 (2.0)	5 (2.7)	1 (0.8)	0.261
None (or medical only)	91 (29.9)	4 (2.2)	87 (73.7)	<0.001

EMS, emergency medical services; LAD, left anterior descending artery; LCx, left circumflex artery; RCA, right circumflex artery; TIMI, thrombolysis in myocardial infarction; PCI, percutaneous coronary intervention; CABG, cardiac artery bypass grafting; POBA, plain old balloon angioplasty.

The AI model test characteristics were superior compared with traditional STEMI millimetre criteria with a sensitivity of 89.2% [95% confidence interval (CI): 84.0–92.9] vs. 68.3% (95% CI: 61.3–74.5), specificity 72.9% (95% CI: 64.2–80.1) vs. 51.7% (95% CI: 42.8–60.5), and accuracy 82.9% (95% CI: 78.3–86.7) vs. 61.8 (95% CI: 56.3–67.1) (*Table 2*). Subgroup analyses were completed for acute culprit lesions with various TIMI flows (see Supplementary material online, *Table S1*). The two methods agreed on the presence or absence of an acutely occluded coronary culprit in 204 cases (67.1%). Disagreement occurred in 100 cases (32.9%) and favoured the AI model (82 vs. 18, odds ratio 4.6, 95% CI 2.7–8.1, *P* < 0.001) (*Table 3*). The AI model demonstrated a significantly higher rate of correct predictions compared with STEMI millimetre criteria (*χ*^2^ = 41.0, *P* < 0.001), indicating that when STEMI and OMI predictions disagree, OMI is more than four-fold more likely to be correct.

**Table 2:** Test characteristics of the artificial intelligence model vs. ST-segment elevation myocardial infarction millimetre criteria

	AI model	STEMI Millimetre criteria
Sensitivity (95% CI)	89.2% (84.0–92.9)	68.3% (61.3–74.5)
Specificity (95% CI)	72.9% (64.2–80.1)	51.7% (42.8–60.5)
Accuracy (95% CI)	82.9% (78.3–86.7)	61.8% (56.3–67.1)
PPV (95% CI)	83.8% (78.1–88.3)	69.0% (62.0–75.3)
NPV (95% CI)	81.1% (72.6–87.4)	50.8% (42.0–59.6)
AUROC	0.884 (0.847–0.921)	N/A

PPV, positive predictive value; NPV, negative predictive value; CI, confidence interval; AUROC, area under the receiver operator characteristic curve.

**Table 3:** Contingency table for the artificial intelligence model vs. ST-segment elevation myocardial infarction millimetre criteria

	STEMI correct prediction	STEMI incorrect prediction	Total	*χ* ^2^	OR (95% CI)	*P*-value
AI correct prediction	170	82	252	41.0	4.6 (2.7–8.1)	<0.001
AI incorrect prediction	18	34	52			
Total	188	116	304			

An OR >1 indicates AI model is more correct on discordant cases. AI, artificial intelligence; *χ*^2^, chi-squared; OR, odds ratio; CI, confidence interval.

**Figure 2:**
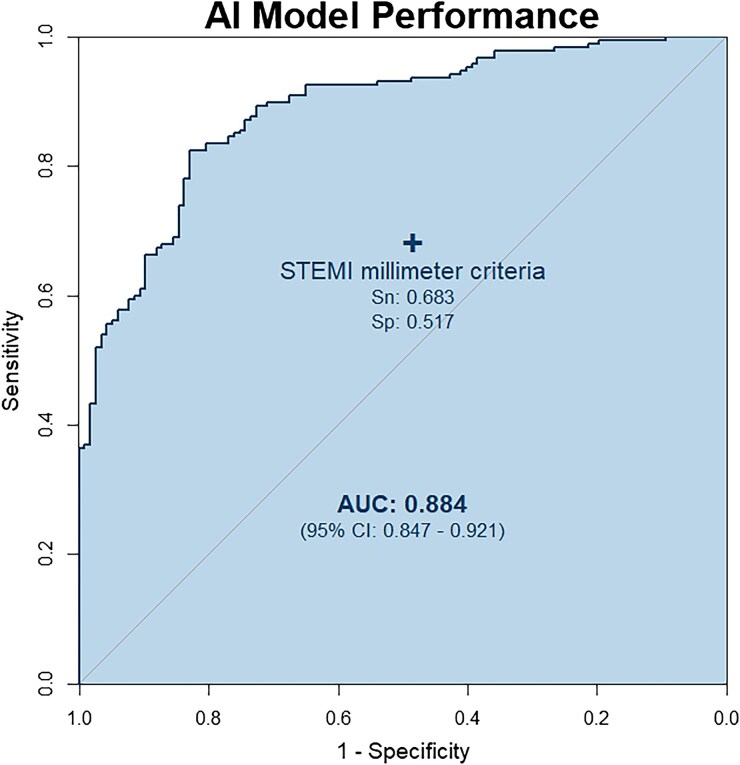
Receiver operator characteristic curve assessing the artificial intelligence model's ability to discriminate cases. The performance of traditional ST-segment elevation myocardial infarction millimetre criteria is marked with a ‘+’ for reference. AUC, area under the curve.

The area under the receiver operator characteristic (AUROC) curve was 0.884 (95% CI: 0.847–0.921) (*Figure 2*).

**Figure 3:**
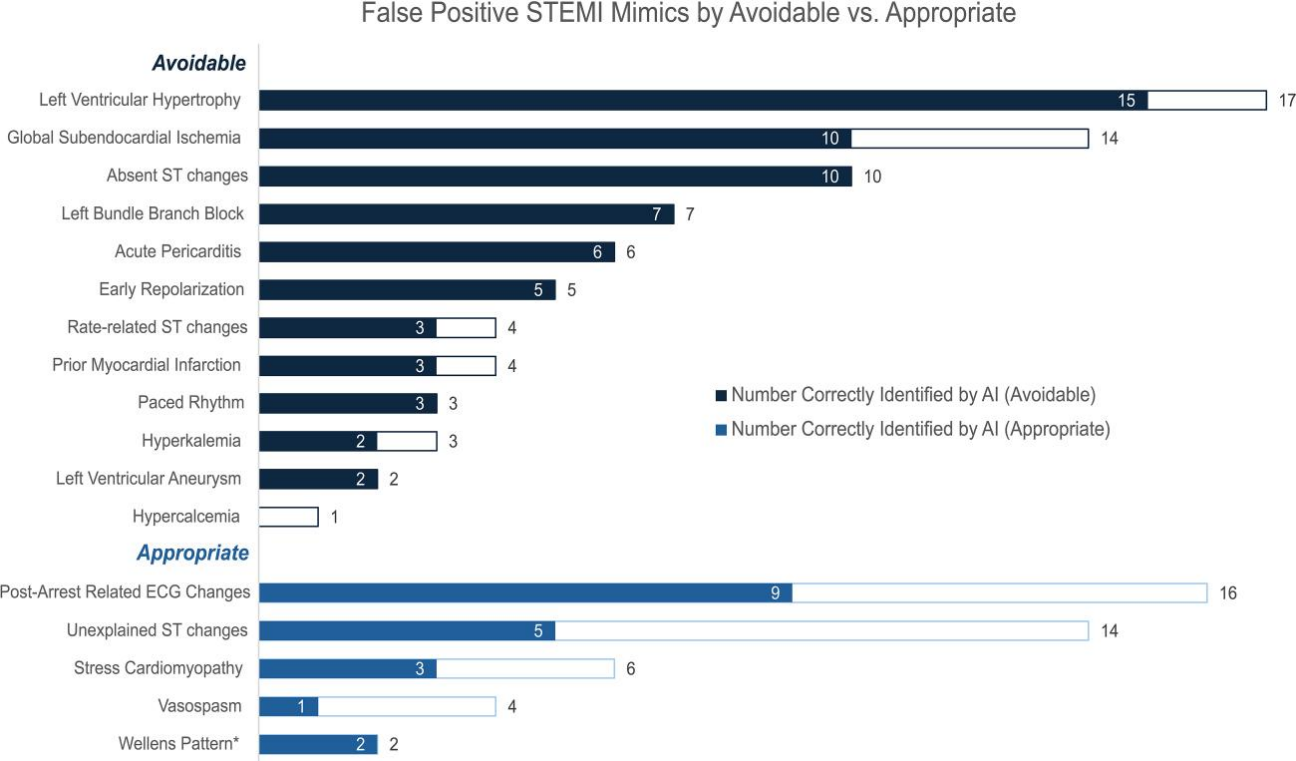
False-positive ST-segment elevation myocardial infarction mimics by avoidable vs. appropriate cardiac catheterization laboratory activations. The shaded region represents false positives correctly identified by the artificial intelligence model. *Two cases with the Wellens pattern were activated after cardiology review.

The most common causes of false-positive STEMI activations were left ventricular hypertrophy (LVH) and post-arrest ECG changes. The AI model correctly identified 86.8% (66/76) of potentially avoidable CCL activations (*Figure 3*). Characteristics of false-negative cases (20; i.e. AI model negative but OMI positive) are included in Supplementary material online, *Table S2*.

## Discussion

Our results are similar to prior studies evaluating the test characteristics of AI for OMI detection on electrocardiography. In a derivation and validation study, Herman *et al*.^22^ used the model to evaluate 3254 ECGs from 2222 patients and found a sensitivity of 80.6% (95% CI: 76.8–84.0) and specificity of 93.7% (95% CI: 92.6–94.8) compared with STEMI millimetre criteria [sensitivity 32.5% (95% CI: 28.4–36.6), and specificity 97.7% (95% CI: 97.0–98.3)]. In a 1-year retrospective cohort of 217 patients, Choi *et al*.^16^ reported the AI model sensitivity and specificity of 86.5 and 82.2%, respectively, compared with STEMI millimetre criteria of 54.1 and 88.7%, respectively.

The specificity in our cohort is less than previously reported. We suspect this difference could be related to our patient cohort (patients activated via the STEMI pathway vs. patients with suspected ACS in the Herman study) and the complexity of cases received at our clinical site. Our tertiary academic centre receives urgent or emergent, complex, high-risk percutaneous, and surgical interventions from around the region, and many transferred patients have already received interventions (e.g. percutaneous or systemic thrombolysis), complicating electrocardiographic and angiographic findings. Furthermore, it is well known that post-arrest ECGs overestimate acutely occlusive episodes because of resuscitation interventions (e.g. epinephrine, defibrillation, etc.).^23–26^ Our cohort had a high proportion of cardiac arrests prior to angiography (53, 17.4%) that were overrepresented in the false-positives group (*Table 1*), diminishing accuracy in this context. In contrast, we found one abstract in which the authors evaluated the AI model’s performance on 160 out-of-hospital cardiac arrest cases and reported high sensitivity, specificity, and positive predictive value (PPV; 88.7, 81.4, and 70.8%, respectively); however, further training and testing in cardiac arrest patients are warranted.^27^

Previous work has described aetiologies for false-positive activations^4,28^ and the aetiologies for our cohort are similar, although the frequencies are different. Of the 118 false-positive activations, 86 (72.9%) were identified by the AI model, and it performed better in cases that were considered avoidable false-positive activations (66/76, 86.8%). The most common aetiology for false-positive activations was LVH, and the AI model performed well in this category, correctly identifying 15 of 17 cases. Traditional STEMI criteria are defined in the absence of LVH,^9^ and while Armstrong *et al*.^29^ derived an algorithm for diagnosing STEMI in the presence of LVH, a routine decision-aid has yet to be validated. Our cohort shows that the AI model could fill this gap as a reliable tool. The model performed well in the setting of left bundle branch block (7/7) and paced rhythms (3/3). The model struggled with post-arrest cases (9/16), cases lacking clear explanation for ST-segment changes (5/14), and vasospasm (1/4). This underscores the evolving nature of ECG features in cardiac arrest, challenges in identifying causes and interpreting reasons for ST-segment changes in complex cases, and difficulty distinguishing OMI from other transient phenomena like vasospasm (the ECG is unable to distinguish the aetiology of transmural ischaemia, only that it exists). It is now well understood that the global subendocardial ischaemic pattern (e.g. multi-lead ST-depression with aVR ST-elevation) can be associated with any condition that causes a supply/demand mismatch (including multi-vessel disease, acute blood loss, sepsis, respiratory failure, tachydysrhythmias, aortic stenosis, pulmonary embolism, stress cardiomyopathy, and cardiac arrest), LVH, or electrolyte derangements like hypokalaemia.^3,30–35^ Subendocardial ischaemia due to ACS was considered a true positive if the pre-specified criteria were met (see Methods). Given the non-specific nature of the global subendocardial ischaemic pattern, it is not surprising that most, but not all, were correctly identified by the AI model (10/14).

The AI model yields a raw score, indicating the likelihood of OMI. For this study, we used a ≥0.50 cut-off—chosen as the best cut-off for obtaining >98% specificity and maximal sensitivity in the population it was trained on. It is possible, however, to adjust the cut-off to improve sensitivity at the expense of worsening specificity (or vice versa) to meet the needs of a particular centre.

Further validation efforts should focus on prospectively obtained results from non-transferred, non-cardiac arrest patients since prior recent interventions (e.g. epinephrine, defibrillation, thrombolysis, PCI, etc.) are subject to complicate ECG interpretation regardless of the source.

### Limitations

This is a single-centre and retrospective study, limiting generalizability and making the results subject to selection and recall bias. There were significant numbers of patients excluded due to the lack of ECG tracings in the electronic medical record (71/454), potentially biasing results. For serial tracings, investigators reviewed charts to determine the tracing that mostly influenced the activation decision; it is possible that not all relevant tracings were entered into the electronic medical record. Our cohort had a high proportion of transfers (20.7%) and emergency medical service arrivals (56.6%), complicating the procurement of initial ECG tracings; consequently, we were unable to reliably determine potential time savings associated with earlier recognition by the AI model. It should be emphasized that a valid comparison between the sensitivities of routine care and the AI model cannot be conducted using this study cohort due to incorporation bias. Specifically, the sensitivity of routine CCL activation cannot be accurately assessed when the dataset is limited solely to cases involving CCL activations.

## Conclusions

The AI model is superior to traditional STEMI millimetre criteria for detecting acutely occluded or flow-limiting coronary lesions and has the potential to reduce false-positive CCL activations. OMI is a clinical diagnosis, and the AI model has the potential to enhance decision-making accuracy but should not be used as a stand-alone test.

## Supplementary Material

ztaf138_Supplementary_Data

## Data Availability

The data underlying this article cannot be shared publicly for the privacy of individuals in the study. The data will be shared on reasonable request to the corresponding author.
