# Supplementary material for: An artificial intelligence model for electrocardiogram detection of occlusion myocardial infarction: a retrospective study to reduce false-positive cath lab activations
Source: Eur Heart J Digit Health. 2025 Dec 2;7(2):ztaf138. doi: 10.1093/ehjdh/ztaf138 (PMC12853124; doi:10.1093/ehjdh/ztaf138)
Supplement: ztaf138_Supplementary_Data [file ztaf138_supplementary_data.zip › Supplemental Tables.docx]

**Supplemental Table 1.** Test Characteristics of the AI model vs STEMI millimeter criteria.

|  |  |  | **Subgroups** | | | | | | | | | |
| --- | --- | --- | --- | --- | --- | --- | --- | --- | --- | --- | --- | --- |
| **Test Characteristic** | **Entire Cohort (N = 304)** | | **Acute Culprit Lesion (191)** | | **Acute Culprit Lesion +  TIMI 0 (123)** | | **Acute Culprit Lesion +  TIMI 0-1 (151)** | | **Acute Culprit Lesion +  TIMI 0-2 (178)** | | **Acute Culprit Lesion +  TIMI 3 (13)** | |
|  | *AI Model* | *STEMI* | *AI Model* | *STEMI* | *AI Model* | *STEMI* | *AI Model* | *STEMI* | *AI Model* | *STEMI* | *AI Model* | *STEMI* |
| **Sensitivity (95% CI)** | 89.2%  (84.0-92.9) | 68.3%  (61.3-74.5) | 86.9%  (81.4-91.0) | 66.5%  (59.5-72.8) | 91.9%  (85.7-95.5) | 70.7%  (62.2-78.0) | 90.1%  (84.3-93.9) | 70.2%  (62.5-76.9) | 87.6%  (82.0-91.7) | 69.1%  (62.0-75.4) | 76.9%  (49.7-91.8) | 30.8%  (12.7-57.6) |
| **Specificity  (95% CI)** | 72.9%  (64.2-80.1) | 51.7%  (42.8-60.5) | 71.7%  (62.8-79.2) | 49.6%  (40.5-58.6) | 53.0%  (45.8-60.2) | 46.4%  (39.3-53.7) | 59.5%  (51.6-66.9) | 49.0%  (41.2-56.9) | 66.7%  (58.1-74.3) | 51.6%  (42.9-60.1) | 35.4%  (30.1-41.0) | 38.1%  (32.8-43.8) |
| **Accuracy  (95% CI)** | 82.9%  (78.3-86.7) | 61.8%  (56.3-67.1) | 81.3%  (76.5-85.2) | 60.2%  (54.6-65.5) | 68.8%  (63.3-73.7) | 56.3%  (50.6-61.7) | 74.7%  (69.5-79.2) | 59.5%  (53.9-64.9) | 78.9%  (74.0-83.2) | 61.8%  (56.3-67.1) | 37.2%  (31.9-42.7) | 37.8%  (32.6-43.4) |
| **PPV  (95 CI%)** | 83.8%  (78.1-88.3) | 69.0%  (62.0-75.3) | 83.8%  (78.1-88.3) | 69.0%  (62.0-75.3) | 57.1%  (50.1-63.8) | 47.3%  (40.2-54.5) | 68.7%  (61.9-74.7) | 57.6%  (50.4-64.5) | 78.8%  (72.6-83.9) | 66.8%  (59.8-73.2) | 5.1%  (2.8-9.0) | 2.2%  (0.8-5.5) |
| **NPV  (95% CI)** | 81.1%  (72.6-87.4) | 50.8%  (42.0-59.6) | 76.4%  (67.5-83.5) | 46.7%  (38.0-55.6) | 90.6%  (83.5-94.8) | 70.0%  (61.3-77.5) | 85.8%  (78.0-91.2) | 62.5%  (53.6-70.6) | 79.2%  (70.6-85.9) | 54.2%  (45.3-62.8) | 97.2%  (92.0-99.0) | 92.5%  (86.4-96.0) |
| **AUROC  (95% CI)** | 0.884 (0.847-0.921) |  |  |  |  |  |  |  |  |  |  |  |

PPV, positive predictive value; NPV, negative predictive value; AUROC, area under the receiver operator characteristic curve; AI, artificial intelligence; TIMI, thrombolysis in myocardial infarction
True positives were defined as an acute culprit lesion with either (A) TIMI flow grade of 0-2 and any positive troponin, or (B) TIMI flow grade of 3 and a very high peak troponin elevation (hs-cTnT ≥ 1,000 ng/L, hs-cTnI ≥ 5000 ng/L, cTnI of > 10.0 ng/mL, or cTnT of > 1.0 ng/mL).

**Supplemental Table 2.** False negative AI cases.

| **Case** | **Age (years)** | **Sex** | **Arrival Method** | **Arrest prior to ECG?** | **AI Raw** | **STEMI Criteria Binary** | **Culprit Artery** | **TIMI Flow** | **Peak Troponin I (pg/mL)** | **Intervention** | **ECG Comments** | **Coronary Angiography Findings** |
| --- | --- | --- | --- | --- | --- | --- | --- | --- | --- | --- | --- | --- |
| 1 | 51 | M | EMS | No | 0.300865395 | TRUE | LAD | 2 | N/A^1^ | PCI | aVL TWI, HATWs | 90% first diagonal ostial stenosis |
| 2 | 70 | F | Inpatient | No | 0.137424922 | TRUE | LAD | 1 | >125,000 | PCI | V1/V2 STE, inferolateral STD | 99% stenosis on mid LAD |
| 3 | 80 | M | Walk-in | No | 0.001080781 | FALSE | RCA | 1 | 8,987 | PCI | inferolateral TWI, LVH | 90% stenosis before PL and PD bifurcation |
| 4 | 63 | M | EMS | Yes | 0.452505033 | FALSE | LM | 2 | N/A^1^ | PCI | LBBB | 90% hazy stenosis dLM |
| 5 | 38 | M | Inpatient | No | 0.006344632 | FALSE | RCA | 0 | 2,245 | POBA | Aslanger pattern | 100% mRCA ISR |
| 6 | 56 | M | Transfer | Yes | 0.42260261 | FALSE | MV | 2 | 92 | PCI | LBBB, lateral STD | pLCx 99%, LM 80%, pLAD 80% |
| 7 | 61 | M | EMS | Yes | 0.044537094 | FALSE | LCx | 0 | 86 | POBA | lateral TWI | 100% thrombotic occlusion of proximal LCx |
| 8 | 25 | M | Inpatient | No | 0.303525126 | TRUE | LAD | 0 | 151,169 | PCI | V4/V4 STE | 100% thrombotic occlusion of the apical LAD from LV thrombus embolization |
| 9 | 80 | M | EMS | Yes | 0.03215854 | FALSE | LAD | 0 | 67,146 | PCI | changes of hyperkalemia | 100% thrombotic occlusion ostial LAD |
| 10 | 64 | M | Transfer | No | 0.271394065 | FALSE | LCx | 0 | 23,922 | PCI | LBBB | 100% acute thrombotic occlusion prox to mid LCx. |
| 11 | 90 | M | Walk-in | Yes | 0.077924643 | TRUE | RCA | 0 | 19,766 | PCI | RBBB, LAFB | 100% RCA occlusion |
| 12 | 62 | M | Transfer | No | 0.003177115 | TRUE | RCA | 2 | 5,269 | PCI | RBBB, LPFB | Hazy thrombus in RCA |
| 13 | 51 | M | EMS | No | 0.00255907 | TRUE | LAD | 0 | 183,731 | PCI | RBBB, LAFB | 100% thrombotic occlusion mid LAD |
| 14 | 79 | M | EMS | No | 0.013663261 | TRUE | RCA | 1 | 3,214 | PCI | RBBB, LAFB | 99% hazy lesion pRCA |
| 15 | 58 | M | EMS | No | 0.037708625 | FALSE | RCA | 0 | 34,345 | PCI | aVL TWI | 100% thrombotic occlusion PLB |
| 16 | 67 | F | EMS | No | 0.024465357 | FALSE | RCA | 2 | 74,194 | PCI | LVH, inferolateral TWI | 95% thrombotic occlusion ostial RCA |
| 17 | 54 | M | Walk-in | No | 0.025963341 | FALSE | LCx | 0 | >125,000 | PCI | early R wave transition | 100% thrombotic occlusion proximal LCx |
| 18 | 55 | F | Transfer | No | 0.269863881 | TRUE | RCA | 3 | 2,318^2^ | None | subtle inferior STE | RCA SCAD |
| 19 | 73 | M | Transfer | Yes | 0.028019622 | FALSE | LAD | 0 | 28,683 | PCI | RBBB | VA ECMO cannulation, LAD culprit |
| 20 | 75 | F | Inpatient | No | 0.280479805 | FALSE | MV | 2 | 3,613 | PCI | SEI pattern | dLM 40-50%, pLAD 80% |

EMS, emergency medical services; HATW, hyperacute T wave; LAD, left anterior descending artery; LAFB, left anterior fascicular block; LBBB, left bundle branch block; LCx, left circumflex artery; LM, left main artery; LPFB, left posterior fascicular block; LV, left ventricle; LVH, left ventricular hypertrophy; PD, posterior descending; PL, posterolateral; PLB, posterolateral branch artery; RBBB, right bundle branch block; RCA, right coronary artery; SCAD, spontaneous coronary artery dissection; SEI, subendocardial ischemia; STD, ST-segment depression; STE, ST-segment elevation; TWI, T wave inversion(s); VA ECMO, veno-arterial extracorporeal membrane oxygenation

*^1^Troponin levels were not available.*

*^2^Case adjudicated to be a true positive despite TIMI 3 flow and failure to reach the prespecified troponin elevation of 5,000 pg/mL because the troponin was not trended to peak and the delta between the two troponin values demonstrated marked upward trend (1,210, drawn 100 minutes apart).*
