# Supplementary figures and images for: An artificial intelligence model for electrocardiogram detection of occlusion myocardial infarction: a retrospective study to reduce false-positive cath lab activations
Source: Eur Heart J Digit Health. 2025 Dec 2;7(2):ztaf138. doi: 10.1093/ehjdh/ztaf138 (PMC12853124; doi:10.1093/ehjdh/ztaf138)

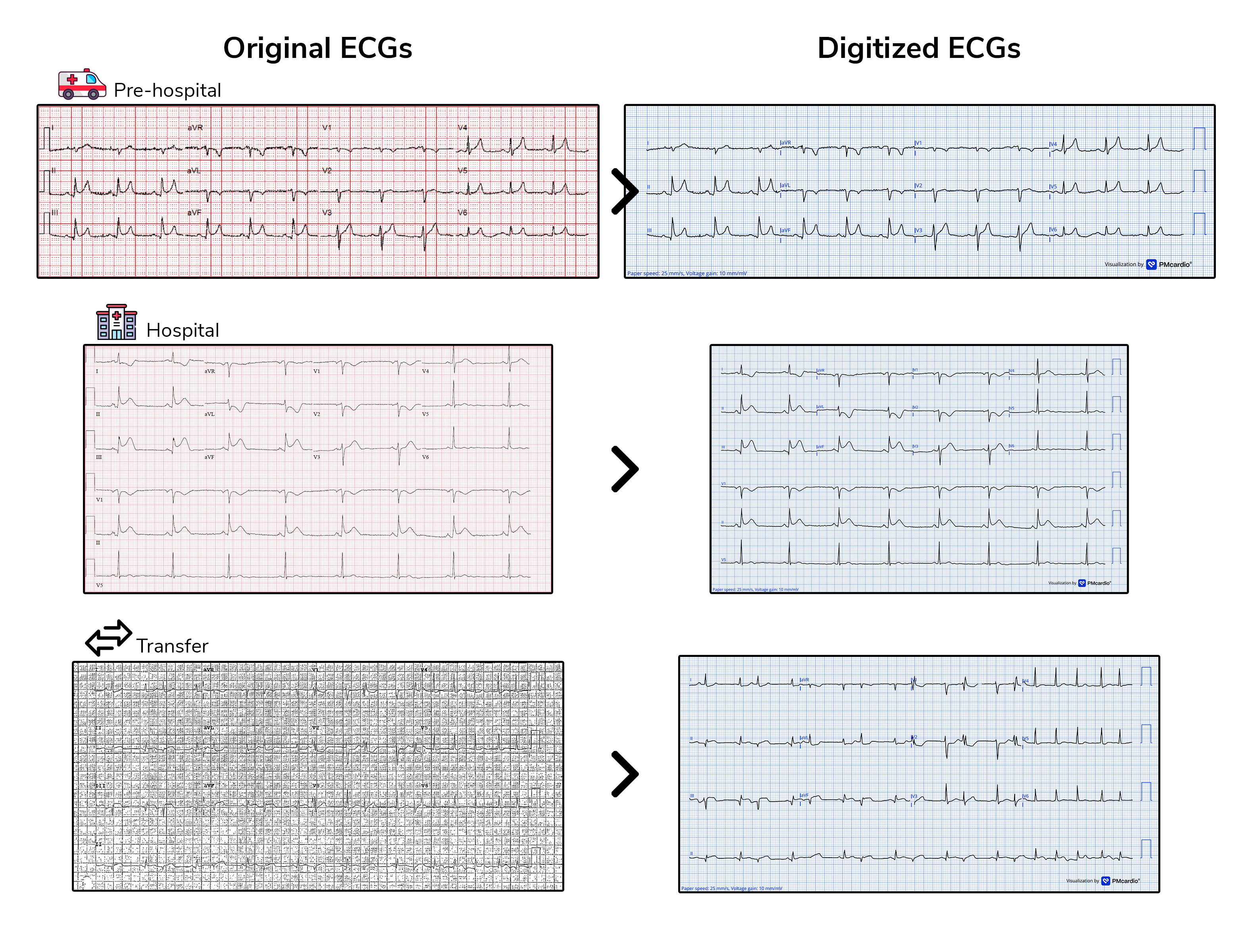

Supplement: ztaf138_Supplementary_Data [file ztaf138_supplementary_data.zip › Supplementary-Figure.jpg]
